# Prevalence, Recurrence, and Incidence of Current Depressive Symptoms among People Living with HIV in Ontario, Canada: Results from the Ontario HIV Treatment Network Cohort Study

**DOI:** 10.1371/journal.pone.0165816

**Published:** 2016-11-01

**Authors:** Stephanie K. Y. Choi, Eleanor Boyle, John Cairney, Evan J. Collins, Sandra Gardner, Jean Bacon, Sean B. Rourke

**Affiliations:** 1 The Institute of Medical Science, Faculty of Medicine, University of Toronto, Toronto, Ontario, Canada; 2 Ontario HIV Treatment Network, Toronto, Ontario, Canada; 3 Dalla Lana School of Public Health, University of Toronto, Toronto, Ontario, Canada; 4 Institute of Sports Science and Clinical Biomechanics, University of Southern Denmark, Odense, Denmark; 5 Department of Psychiatry, Faculty of Medicine, University of Toronto, Toronto, Ontario, Canada; 6 Department of Family Medicine, McMaster University, Hamilton, Ontario, Canada; 7 Infant and Child Health Lab, McMaster University, Hamilton, Ontario, Canada; 8 Department of Clinical Epidemiology and Biostatistics, McMaster University, Hamilton, Ontario, Canada; 9 CanChild Centre for Childhood Disability Research, McMaster University, Hamilton, Ontario, Canada; 10 Offord Centre for Child Studies, McMaster University, Hamilton, Ontario, Canada; 11 Department of Psychiatry and Behavioural Neuroscience, McMaster University, Hamilton, Ontario, Canada; 12 Centre for Addiction and Mental Health, Toronto, Ontario, Canada; 13 Institute for Clinical Evaluative Sciences, Toronto, Ontario, Canada; 14 Faculty of Kinesiology and Physical Education, University of Toronto, Toronto, Ontario, Canada; 15 University Health Network, Toronto, Ontario, Canada; 16 St. Michael’s Hospital, Toronto, Ontario, Canada; British Columbia Centre for Excellence in HIV/AIDS, CANADA

## Abstract

**Introduction:**

Current studies of depression among people living with HIV focus on describing its point prevalence. Given the fluctuating nature of depression and its profound impacts on clinical and quality-of-life outcomes, this study aimed to examine the prevalence, recurrence and incidence of current depressive symptoms and its underlying catalysts longitudinally and systematically among these individuals.

**Methods:**

We conducted a prospective cohort study between October 1, 2007 and December 31, 2012 using longitudinal linked data sources. Current depressive symptoms was identified using the Centre for Epidemiologic Studies Depression Scale or the Kessler Psychological Distress Scale, first at baseline and again during follow-up interviews. Multivariable regressions were used to characterize the three outcomes.

**Results:**

Of the 3,816 HIV-positive participants, the point prevalence of depressive symptoms was estimated at 28%. Of the 957 participants who were identified with depressive symptoms at baseline and who had at least two years of follow-up, 43% had a recurrent episode. The cumulative incidence among 1,745 previously depressive symptoms free participants (at or prior to baseline) was 14%. During the five-year follow-up, our multivariable models showed that participants with greater risk of recurrent cases were more likely to feel worried about their housing situation. Participants at risk of developing incident cases were also likely to be younger, gay or bisexual, and unable to afford housing-related expenses.

**Conclusions:**

Depressive symptoms are prevalent and likely to recur among people living with HIV. Our results support the direction of Ontario’s HIV/AIDS Strategy to 2026, which addresses medical concerns associated with HIV (such as depression) and the social drivers of health in order to enhance the overall well-being of people living with or at risk of HIV. Our findings reinforce the importance of providing effective mental health care and demonstrate the need for long-term support and routine management of depression, particularly for individuals at high risk.

## Introduction

Depression affects up to half of people living with HIV, a prevalence that is two to four times higher than that found in the general population [1]. Over 50% of people living with HIV and depression do not receive treatment for their depression [2–9], and this failure to treat contributes to significant negative clinical and quality-of-life outcomes [10–14].

Growing evidence supports a bi-directional relationship between HIV and depression involving a number of biological, psychosocial and social factors [1,14–16]. The persistent viral presence in the central nervous system may release toxic viral proteins that induce depression-like symptoms [17,18]; people living with HIV may possess a negative self-image or experience stigma [1,15,19–21]; and people living with HIV are more likely to struggle with stressors such as financial insecurity and unstable housing [22–25]. Recent reviews also suggest that people who suffer from severe mental illnesses (including depression) and/or co-occurring substance use disorder are more likely to engage in risky sexual behaviour, thereby elevating their risk of HIV acquisition [26–34].

To date, most studies about the prevalence of depression among people living with HIV have used cross-sectional designs [1,15]. Six studies have documented the incidence [35–38] and persistence (or recurrence) [39,40] of depression over time among people living with HIV. In Canada, information describing the epidemiology of depression among people living with HIV is scarce. In Canada, information describing the epidemiology of depression among people living with HIV is scarce. There have been two small convenience sample studies describing the prevalence of depression among people living with HIV. Williams et al. (2005), employing a small convenience sample of 297 individuals, described the prevalence of depressive symptoms at 54% among people living with HIV based on a self-report screening instrument [41]. Logie, James, Tharao, and Loutfy (2013), employing a sample of 173 Africa, Caribbean, and Black women, described the prevalence of depressive symptoms as 64% [42]. Thus, the epidemiology of this condition is not yet well documented in Canada.

Given the fluctuating nature of depression over the life span and its profound impacts on clinical and quality-of-life outcomes, our study aimed to examine the prevalence, recurrence, and incidence of current depressive symptoms longitudinally and systematically among people living with HIV. We also characterized these three outcomes by HIV-positive participants’ socio-demographic characteristics, housing and neighbourhood conditions, substance-use behaviours and health status over a five-year follow-up period. Understanding change in the burden of depressive symptoms and the underlying catalysts of the condition from a longitudinal perspective would be important to program planners, policy-makers, and health care providers when planning and implementing effective mental-health programs and interventions for people living with HIV.

## Materials and Methods

### Study Design and Data Sources

We conducted a prospective cohort study between October 1, 2007 and December 31, 2012 by linking unique encoded identifiers from the Ontario HIV Treatment Network Cohort Study (OCS) with national and provincial administrative health databases held at the Institute for Clinical Evaluative Sciences (ICES). Details about the linked data source have been provided in a recent study [9]. We have obtained ethics approvals for the use of the linked data from the University of Toronto, the institutional review board at Sunnybrook Health Sciences Centre, and participating HIV clinics across Ontario (i.e., the Institutional Review Board at Sunnybrook Health Sciences Centre, Ottawa Health Science Network Research Ethics Board, The University of Western Ontario Research Ethics Board for Health Sciences Research involving Human Subjects, St. Michael's Hospital Research Ethics Board, the Research Ethics Board of Health Sciences North, Sunnybrook Health Sciences Centre Research Ethics Board, University Health Network Research Ethics Board, and Windsor Regional Hospital Research Ethics Board).

The OCS is a multi-site HIV cohort, described in detail in a previous publication [43]. HIV-positive participants who received care were recruited from HIV specialty care clinics in Ontario. Clinical nurses and assistants interviewed the HIV-positive participants during regular clinical appointments [43]. The OCS data source contains participants’ medical records from chart abstractions, HIV viral load/HIV antigen test records provided by Public Health Ontario Laboratories, socio-demographics, and psychosocial and behavioural data [43]. The frequency of follow-up for each HIV-positive participant depended on the frequency of his/her clinical appointments. Most HIV-positive participants completed follow-up interviews annually, while 3.5% of the sample completed interviews more than once a year. We defined baseline as the time when the HIV-positive participants completed their first interview. The median number of interviews completed during our study period by each participant was three (interquartile range: 2–4).

We used four administrative health databases to obtain additional information about participants’ past diagnoses of depression, other psychiatric disorders and death records. In Canada, according to the Canada Health Act, the publicly funded universal health care system covers medical and hospital services provided by physicians for insured residents of Canada, and these databases capture information for all publicly-funded services.

1. The *Ontario Health Insurance Plan (OHIP)* database contains billing records for all insured services claimed by physicians and other health professionals.
2. The *Canadian Institute for Health Information Discharge Abstracts Database (DAD)* contains abstracts of all discharges from acute, chronic and rehabilitation inpatient facilities [44].
3. The *National Ambulatory Care Reporting System (NACRS)* captures all emergency department visits [45].
4. The *Registered Persons Database (RPDB)* captures death records of everyone insured under OHIP.

### Study Participants

HIV-positive participants were included in the analysis if they were 16 years or older, had a valid OHIP number to link to administrative databases, had completed their baseline interview between October 1, 2007 and December 31, 2012, and had one measure of their current level of depressive symptoms available.

### Determining Depressive Symptoms

Current depressive symptoms (i.e., within the past month) was identified using two short screening instruments. Due to resource-constraints in several HIV clinics, 61% of HIV-positive participants were administered the 10-item Kessler Psychological Distress Scale (K_10_) and the rest were administered the 20-item Centre for Epidemiologic Depression Scale (CES-D_20_). Diagnostic accuracy and reliability of the two screening instruments were verified against Diagnostic and Statistical Manual of Mental Disorders, Fourth Edition, Text Revision (DSM-IV-TR) criteria for a major depression diagnosis from a recent study conducted in a sample of HIV-positive participants [46]. To identify current depressive symptoms, we used a cut-point of 22 (sensitivity: 0.97; specificity: 0.81) for K_10_ [46] and a cut-point of 23 (sensitivity: 1.0; specificity: 0.87) for CES-D_20_ [46]. The CES-D_20_ and K_10_ demonstrate good inter-rater agreement (Cohen’s Kappa Statistic = 0.79) when compared with the DSM-IV-TR criteria for a diagnosis of major depression [46]. Current depressive symptoms was measured at baseline and at each follow-up. CES-D_20_ and K_10_ have been adopted to identify depressive symptoms in general population [47–49], HIV-positive individuals [50,51], and individuals with chronic illness [52].

A history of depression was defined as having an International Statistical Classification of Diseases 9^th^ revision (ICD-9) or 10^th^ revision (ICD-10) diagnostic code (Table 1) in the OHIP, DAD or NACRS databases from the earliest available record until one year prior to baseline [44,45]. We allowed one year as a washout period to avoid any overlap between current depressive symptoms and past diagnosis of depression [53].

**Table 1:** International Statistical Classification of Diseases 9^th^ (ICD-9) or 10^th^ (ICD-10) revision Diagnostic Codes for Identifying History of Depression.

ICD-9 diagnostic codes		ICD-10 diagnostic codes	
296.2	Major depressive disorder single episode	F32.0	Mild depressive episode
309.0	Adjustment disorder with depressed mood	F33.0	Recurrent depressive disorder, current episode mild
296.3	Major depressive affective disorder, recurrent episode	F32.1	Moderate depressive episode
309.1	prolonged depressive reaction	F33.1	Recurrent depressive disorder, current episode moderate
311	Depressive disorder, not elsewhere classified	F32.2	Severe depressive episode without psychotic symptoms
300.4	Dysthymic disorder	F32.3	Severe depressive episode with psychotic symptoms
		F33.2	Recurrent depressive disorder, current episode severe without psychotic symptoms
		F33.3	Recurrent depressive disorder, current episode severe with psychotic symptoms
		F33.4	Recurrent depressive disorder, currently in remission
		F33.8	Other recurrent depressive disorders
		F33.9	Recurrent depressive disorder, unspecified
		F32.8	Other depressive episodes
		F32.9	Other depressive episodes
		F38.0	Other single mood disorders
		F38.1	Other recurrent mood disorders
		F38.8	Other specified mood disorders

**Fig 1:**
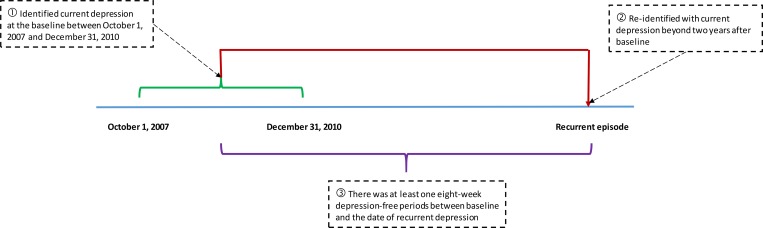
Illustration for Definition of Recurrent Depressive Symptoms.

A *prevalent case* was defined as a participant with current depressive symptoms at baseline. An *incident case* was defined as a depressive symptoms-free participant (at or prior to baseline) having their first episode of depressive symptoms during the five-year follow-up period. A *first recurrent case* was defined as a participant who was identified with current depressive symptoms at baseline, re-identified with current depressive symptoms beyond two years after baseline, and for whom there was at least one eight-week depression-free periods between baseline and the date of recurrent depressive symptoms (Fig 1) [54,55]. To identify a depression-free period, we looked for depression-related diagnostic codes in the OHIP, DAD, or NACRS databases between interview dates.

### Mental Disorder Co-Morbidities

We measured other mental disorder co-morbidities (except for depression-related conditions) according to the sixteen ICD-9 disease sub-categories [56]. This diagnostic information was obtained from the OHIP database from the earliest available record [56].

### Individual and Contextual Explanatory Variables

We selected individual explanatory variables and contextual explanatory variables (housing status, participants’ perception of their neighbourhood) known to be associated with depressive symptoms. More details about these explanatory variables are provided in S1 Appendix.

### Statistical Analysis

All statistical tests were two-sided with statistical significance defined as a p-value<0.05. Analyses were performed using STATA MP v. 13.1 [57]. Our analyses were conducted at the ICES.

**Fig 2:**
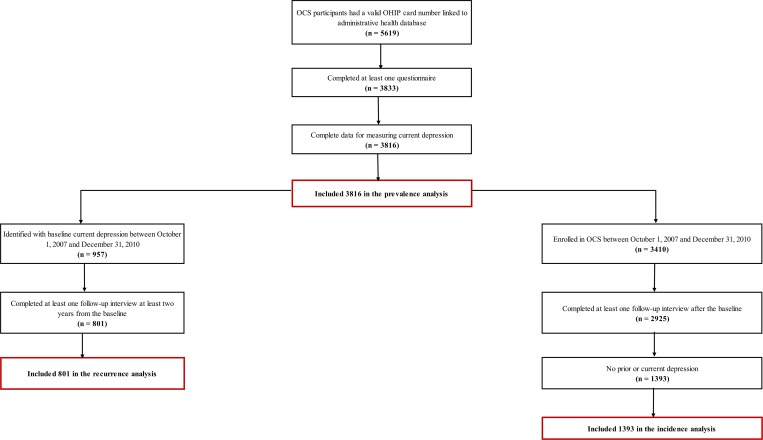
Participant Flow Chart for Development of Prevalence, Recurrence, and Incidence Cohorts.

We described overall and explanatory variable-specific prevalence (point and period), recurrence, and incidence for current depressive symptoms. Point prevalence was calculated as the number of cases with current depressive symptoms at baseline. The denominator in point prevalence calculations included HIV-positive participants who had completed their baseline interview between October 1, 2007 and December 31, 2012, and who had one measure for identifying current depressive symptoms (Fig 2). The period prevalence rate was calculated as the number of cases with depressive symptoms per 100 person-years during the five-year follow-up period. The denominator in period prevalence rate calculations was person-years of HIV-positive participants included in the point prevalence calculation (Fig 2). The recurrence rate was calculated as the number of first recurrent cases with depressive symptoms per 100 person-years during the five-year follow-up period among participants identified with current depressive symptoms at baseline (Fig 2). The denominator in recurrence calculations were person-years of HIV-positive participants who had completed their baseline interview between October 1, 2007 and December 31, 2010, and who had identified with current depressive symptoms at baseline (Fig 2). We allowed at least two years to observe recurrent cases until the end of our study period (Fig 2). The incidence rate was calculated as the number of incident cases with depressive symptoms per 100 person-years during the five-year follow-up period among participants who were depressive symptom-free at baseline and previously (Fig 2). The denominator in incidence calculations were person-years of HIV-positive participants who had completed their baseline interview between October 1, 2007 and December 31, 2011, and who were depressive symptom-free at or prior to the baseline (Fig 2). We allowed at least one year to observe incident cases until the end of our study period (Fig 2). We used Wald test for bivariable point prevalence comparison, and a Logrank test for bivariable period prevalence, incidence, and recurrence rates comparison.

We also used frequency and proportion to determine how many participants had mental disorder co-morbidities in addition to current depressive symptoms at baseline. We used Chi-square or Fisher exact test for bivariable proportion comparison by the participants’ current status of depressive symptoms at baseline.

Several explanatory multivariable models were constructed to examine associations between potential explanatory variables and the point prevalence, recurrence and incidence of current depressive symptoms. Explanatory variables entered into and kept in the explanatory multivariate models were based on evidence from prior studies [38,58–61]. When an explanatory variable did not have a priori evidence, we used a backward selection method [62–64]. This was done by calculating the p value thresholds of the Akaike information criteria (AICs) or Bayesian information criteria (BICs) for two nested models. A p-value threshold of either <0.00408 or <0.2 would provide the minimal BIC/AIC when deciding if an explanatory variable without a priori literature should be kept in the model. Because there is a wide gap between these two p-value thresholds, we considered two more p-value thresholds: p< 0.15 and p< 0.10. At each step of the backward selection procedure, the p value threshold was used to determine if it was appropriate to keep the explanatory variable. This process was done for all of the p value thresholds, separately. The final set of explanatory variables was selected based on the lowest AICs and BICs of the full model (include all explanatory variables) and the final models according to the four p-value criteria [62–64]. To further examine uncertainty in the model building procedure, we generated 1,000 bootstrapped samples from our original dataset to examine the probability for each explanatory variable (those without prior evidence) to be retained in the final model.

As we discussed in the methods section, K_10_ was administered in several HIV clinics with resources constraints, it is possible that the characteristics of participants differed between clinics. In each of our final explanatory models, we controlled for differences between the CES-D_20_ and K_10_ as well as the residual difference of participants’ characteristics by the clinic type.

We first constructed modified Poisson regression models with robust error variance to examine factors associated with point prevalence [65].

Second, we constructed Cox proportional hazard regression models to examine factors associated with first recurrent depressive symptoms during the five-year follow-up period among HIV-positive participants with current depressive symptoms. In the final model, we also controlled for the use of antidepressants as identified in the participants’ medical records from clinical abstractions. The definition of antidepressants was based on the first line of antidepressants for managing depression in adults recommended by the Canadian Network for Mood and Anxiety Treatments (CANMAT) Clinical guidelines [66].

Third, we constructed Cox proportional hazard regression models to examine factors associated with the incidence of depressive symptoms among HIV-positive participants who had no history of depression at or prior to baseline.

For each Cox model, we tested proportionality assumptions by Schoenfeld residuals against time for each explanatory variable and interactions with linear time and natural log of time. When the variable did not meet the proportionality assumptions, our final multivariable Cox proportional hazard model was stratified by this variable that did not meet the assumptions.

Adjusted relative risk (aRR) and 95% confidence intervals (CIs) were reported for the association between each explanatory variable and point prevalence outcome. Adjusted hazard ratio (aHR) were reported for the association between each explanatory variable and incidence and recurrence outcomes.

## Results

We included 3,816 HIV-positive participants at baseline in our final analysis. The median age of participants was 46 years (Interquartile range: 39–52), and 17% were female. Table 2 contains the baseline characteristics of our sample overall and by instrument type. Briefly, 66% were gay or bisexual, 45% had annual household incomes below $40K CAD, 41% were current smokers and 39% had a history of depression. We noted a number of difference in participants by instrument types. For example, participants who completed the K_10_ (as opposed to the CES-D_20_) were more likely to be female (57 vs. 43%, p-value = 0.01633), more likely to have difficulty in affording housing-related expenses (53 vs. 47%, p<0.0001), more likely to have a sense of belonging to their neighbourhoods (57 vs. 43%, p-value<0.0001), and more likely to have control over their housing situation (58 vs. 42%, p-value<0.0001). We therefore included a variable to control for differences between instrument types in our final models.

**Table 2:** Baseline Characteristics of the Samples (N = 3816).

Characteristics	Total		By Instrument Types ^a^				
			K_10_		CES-D_20_		p-value
	(N = 3,816)		(N = 2346)		(N = 1470)		
	N	%	N	%	N	%	
**Demographics**							
Age							**0.004090**
16–29 years	273	(7%)	180	(66%)	93	(34%)	
30–39 years	651	(17%)	426	(65%)	225	(35%)	
40–49 years	1492	(39%)	929	(62%)	563	(38%)	
≥ 50 years	1244	(33%)	726	(58%)	518	(42%)	
Gender							**0.01633**
Female	646	(17%)	366	(57%)	280	(43%)	
Male	3169	(83%)	1979	(62%)	1190	(38%)	
Sexual orientation							0.1242
Gay, lesbian, or bisexual	2,532	(66%)	1585	(63%)	947	(37%)	
Heterosexual	1262	(33%)	747	(59%)	515	(41%)	
Marital status							0.5413
Married / living with partners	1,517	(40%)	940	(62%)	577	(38%)	
Single, separated/divorced, or widowed	2287	(60%)	1397	(61%)	890	(39%)	
Ethnic identity							**< 0.0001**
European descent	3,217	(84%)	1992	(62%)	1225	(38%)	
First Nation, Metis, or Inuit	383	(10%)	295	(77%)	88	(23%)	
African, Caribbean, Asian, or Latin American	215	(6%)	59	(27%)	156	(73%)	
Immigration status							**< 0.0001**
Canadian immigrant	1,110	(29%)	510	(46%)	600	(54%)	
Canadian born	2697	(71%)	1835	(68%)	862	(32%)	
**Socio-economic Status**							
Current employment status							0.2127
Employed	1799	(47%)	1079	(61%)	702	(39%)	
Unemployed	363	(10%)	219	(60%)	144	(40%)	
Student/Retired	329	(9%)	204	(62%)	125	(38%)	
Recipient of Ontario Disability Support Program	1,312	(34%)	814	(62%)	498	(38%)	
Educational attainment							
Completed high school or less	1,245	(33%)	774	(62%)	471	(38%)	0.5418
More than high shcool	2571	(67%)	1572	(61%)	999	(39%)	
Annual household income (CAD) before withholding taxes/benefits							**< 0.0001**
< $20,000	995	(26%)	599	(60%)	396	(40%)	
$20,000 to $39,999	709	(19%)	418	(59%)	291	(41%)	
$40,000 to $49,999	483	(13%)	298	(62%)	185	(38%)	
≥ $50,000	1252	(33%)	752	(60%)	500	(40%)	
**Housing and Neighbourhood Conditions**							
Difficulty in affording housing-related expenses ^b^							**< 0.0001**
Yes	889	(23%)	469	(53%)	420	(47%)	
No	2552	(67%)	1509	(59%)	1043	(41%)	
Worry about being evicted ^c^							**< 0.0001**
Yes	592	(16%)	309	(52%)	283	(48%)	
No	2863	(75%)	1677	(59%)	1186	(41%)	
Having control over their housing situation ^c^							**<0.0001**
Yes	2881	(76%)	1676	(58%)	1205	(42%)	
No	575	(15%)	310	(54%)	265	(46%)	
**Sense of** belonging to neighbourhood ^c^							**< 0.0001**
Yes	2513	(66%)	1435	(57%)	1078	(43%)	
No	943	(25%)	551	(58%)	392	(42%)	
Perceive good location of their home ^c^							**< 0.0001**
Yes	2901	(76%)	1672	(58%)	1229	(42%)	
No	555	(15%)	310	(54%)	265	(46%)	
**Harmful Behaviours**							
Recreational drug use in past 6 months							**< 0.0001**
Yes	807	(21%)	561	(70%)	246	(30%)	
No	3006	(79%)	1784	(59%)	1222	(41%)	
Prior diagnosis of alcohol abuse ^d^							**0.02396**
Yes	435	(11%)	289	(66%)	146	(34%)	
No	3381	(89%)	2057	(61%)	1324	(39%)	
Current smoker							**< 0.0001**
Yes	1,567	(41%)	1056	(67%)	511	(33%)	
No	2237	(59%)	1278	(57%)	959	(43%)	
**Health Status**							
Current depressive symptoms							**< 0.0001**
Yes	1070	(28%)	742	(69%)	328	(31%)	
No							
History of depression ^e^							**< 0.0001**
Yes	1,504	(39%)	988	(66%)	516	(34%)	
No	2312	(61%)	1358	(59%)	954	(41%)	
Physical component of SF-12, median (25^th^ to 75^th^ percentile)	52	(42–57)	52	(41–57)	52	(43–56)	0.3526
Charlson co-morbidity index ≥ 1							0.3245
Yes	966	(25%)	581	(60%)	385	(40%)	
No	2850	(75%)	1765	(62%)	1085	(38%)	
Non-suppressed recent viral loads (> 50 *μL*) (in past 6 months)							0.2999
Yes	1080	(28%)	678	(63%)	402	(37%)	
No	2736	(72%)	1668	(61%)	1068	(39%)	
Years since HIV diagnosis, median (25^th^ to 75^th^ percentile)	11	(5–17)	11	(5–17)	11	(5–17)	0.3020

^a^ There are two instruments for identifying current depressive symptoms administered by clinic nurses and assistant during the participant’s regular clinical appointments. Due to constraints on human resources and time in several HIV clinics, 61% of HIV-positive participants were administered the 10-item Kessler Psychological Distress Scale (K_10_) and 39% were administrated the 20-item Centre for Epidemiologic Studies Depression Scale (CES-D_20_). Full details of the cohort can be found on the study website: http://www.ohtncohortstudy.ca/

^b^ Difficulty in affording house-related expenses was defined as a participant’s self-reported “Very difficult” or “Fairly difficult” for the following question: *“Considering your household income*, *how difficult is it for you to meet your monthly housing-related costs*?*(Housing costs include rent/mortgage*, *property taxes and utilities only)*.*”*

^c^ A 5-point Likert scale (strongly agree to strongly disagree) was used. We dichotomized their response into “yes” (strongly agree/agree) and “no” (neutral/disagree/strongly disagree).

^d^ Addiction to alcohol was defined as a diagnostic code of alcohol dependence/abuse in OHIP (ICD-9: 303) or in main diagnosis of DAD and NACRS (ICD-9-CM: 303; ICD-10-CA: F10), from the earliest available records to a day before the baseline.

^e^ History of depression was defined as having a past depression-related diagnosis in OHIP records (OHIP ICD-9: 296 and 311), from the earliest available records to a year before baseline.

### Prevalence of Current Depressive Symptoms

We included 3,816 HIV-positive participants in our prevalence estimation of current depressive symptoms (Fig 2 & Table 3). When we compared the characteristics of the participants included in the prevalence sub-cohort to the remainder of the cohort, we found that participants were slightly younger (mean age: 53 v. 56 years; p-value: <0.0001) and more likely to be female (16.8 v. 10.5%; p-value: <0.0001).

**Table 3:** Point Prevalence (PP), Period Prevalence Rate (PPR), Recurrence Rate (RR) Incidence Rate (IR) of Current Depressive Symptoms by Explanatory Variables among HIV-positive Participants.

	Prevalence						Recurrence Rate			Incidence Rate		
	Point Prevalence			Period Prevalence Rate								
	(N = 3816)			(N = 3816)			(N = 957)			(N = 1,745)		
	PP	95% CI	p-value	PPR	95% CI	p-value	RR	95% CI	p-value	IR	95% CI	p-value
**Overall**	28%	(27, 29)		13.5	(12.8, 14.2)		11.9	(10.8, 13.2)		4.5	(3.9, 5.1)	
**Demographics**												
Age			**<0.0001**			**<0.0001**			0.05319			**<0.0001**
16–29 years	30%	(25, 36)		19.3	(16.1, 23.2)		7.4	(4.5, 12.3)		7.2	(4.7, 10.9)	
30–39 years	34%	(30, 37)		17.4	(15.6, 19.6)		8.0	(6.1, 10.4)		6.3	(4.7, 8.4)	
40–49 years	30%	(27, 32)		14.6	(13.5, 15.8)		12.1	(10.4, 14.1)		4.4	(3.5, 5.5)	
≥ 50 years	22%	(20, 24)		10.0	(9.1, 11.0)		15.3	(13.1, 17.9)		3.7	(3.0, 4.6)	
Gender			**<0.0001**			**<0.0001**			0.5826			**0.003427**
Female	37%	(33, 40)		18.8	(16.8, 20.9)		11.3	(9.1, 14.1)		6.1	(4.6, 8.1)	
Male	26%	(25, 28)		12.6	(11.9, 13.3)		12.1	(10.8, 13.4)		4.2	(3.6, 4.8)	
Sexual orientation			**<0.0001**			**<0.0001**			0.8231			0.1463
Gay, lesbian, or bisexual	26%	(24, 27)		12.4	(11.6, 13.2)		12.4	(11.0, 14.0)		4.3	(3.7, 5.1)	
Heterosexual	33%	(30, 36)		16.0	(14.7, 17.4)		11.2	(9.5, 13.2)		4.8	(3.9, 6.0)	
Marital status			**<0.0001**			**<0.0001**			0.06293			**0.000348**
Married / living with partners	22%	(20, 24)		10.5	(9.6, 11.4)		11.0	(9.3, 13.2)		3.4	(2.8, 4.3)	
Single, separated/divorced, or windowed	32%	(30, 34)		15.6	(14.7, 16.6)		12.3	(11.0, 13.9)		5.4	(4.6, 6.3)	
Ethnic identity						**<0.0001**			**0.03283**			**0.02803**
European descent	27%	(26, 29)	**0.000844**	13.0	(12.3, 13.8)		12.3	(11.0, 13.6)		4.3	(3.8, 4.9)	
First Nation, Metis, or Inuit	37%	(32, 42)		18.7	(16.2, 21.5)		11.8	(9.0, 15.4)		6.4	(4.4, 9.3)	
African, Caribbean, Asian, or Latin American	27%	(21, 33)		11.8	(9.3, 14.9)		7.5	(4.4, 12.7)		3.7	(1.7, 8.3)	
Canadian immigration status			0.8150			0.1627			0.2280			0.7249
Yes	27%	(25, 30)		13.1	(11.9, 14.5)		12.3	(10.4, 13.2)		4.7	(3.8, 5.8)	
No	28%	(27, 30)		14.1	(13.3, 15.0)		11.7	(10.3, 14.8)		4.4	(3.7, 5.1)	
**Socio-economic Status**												
Current employment status			**<0.0001**			**<0.0001**			**0.008041**			**<0.0001**
Employed	16%	(14, 17)		7.9	(7.2, 8.7)		11.0	(9.1, 13.2)		3.2	(2.7, 3.9)	
Unemployed	37%	(32, 42)		18.5	(15.9, 21.6)		6.6	(4.3, 10.0)		4.4	(2.7, 7.4)	
Student/Retired	16%	(12, 19)		7.5	(6.1, 9.2)		14.8	(9.9, 22.1)		2.9	(1.9, 4.6)	
Recipient of Ontario Disability Support Program	45%	(43, 48)		23.5	(21.9, 25.2)		13.0	(11.5, 14.7)		8.6	(7.1, 10.4)	
Educational attainment						**<0.0001**			0.9028			**0.000516**
Completed high school or less	36%	(33, 39)	**<0.0001**	17.5	(16.1, 18.9)		11.1	(9.4, 13.0)		6.0	(4.8, 7.4)	
More than high school	24%	(23, 26)		11.7	(11.0, 12.5)		12.5	(11.1, 14.2)		3.9	(3.3, 4.6)	
Annual household income (CAD) before withholding taxes/benefits			**<0.0001**			**<0.0001**			0.1647			**<0.0001**
< $20,000	42%	(39, 45)		22.0	(20.2, 23.8)		12.5	(10.8, 14.5)		8.3	(6.7, 10.4)	
$20,000 to $39,999	32%	(28, 35)		15.5	(13.9, 17.4)		13.4	(11.0, 16.4)		4.7	(3.5, 6.4)	
$40,000 to $49,999	19%	(16, 23)		9.9	(8.4, 11.6)		10.7	(7.7, 14.9)		4.0	(2.8, 5.7)	
≥ $50,000	16%	(14, 18)		7.6	(6.8, 8.5)		10.8	(8.5, 13.6)		2.8	(2.1, 3.5)	
**Housing and Neighbourhood Conditions**												
Difficulty in affording housing-related expenses ^a^			**<0.0001**			**<0.0001**			**0.002721**			**<0.0001**
Yes	45%	(41, 48)		26.2	(24.0, 28.1)		14.5	(12.5, 16.9)		9.7	(7.8, 12.0)	
No	21%	(20, 23)		10.5	(9.8, 11.3)		13.1	(11.5, 14.8)		3.4	(2.9, 4.0)	
Worry about being force out from their home ^b^			**<0.0001**			**<0.0001**			**0.000306**			**<0.0001**
Yes	48%	(44, 52)		28.2	(25.6, 31.2)		15.9	(13.4, 18.9)		11.1	(8.6, 14.3)	
No	23%	(22, 25)		11.5	(10.8, 12.2)		12.8	(11.4, 14.4)		3.7	(3.2, 4.3)	
Having control over their housing situation ^b^			**<0.0001**			**<0.0001**			**0.000134**			**<0.0001**
Yes	23%	(22, 25)		11.6	(10.9, 12.3)		12.9	(11.5, 14.4)		4.0	(3.5, 4.7)	
No	48%	(44, 52)		28.6	(25.8, 31.6)		16.0	(13.4, 19.1)		7.0	(5.4, 9.2)	
Sense of belonging to neighbourhood ^b^			**<0.0001**			**<0.0001**			**0.001541**			**<0.0001**
Yes	22%	(21, 24)		11.0	(10.3, 11.8)		13.3	(11.8, 15.1)		3.8	(3.2, 4.4)	
No	40%	(37, 43)		23.4	(21.4, 25.4)		14.2	(12.2, 16.6)		6.9	(5.5, 8.5)	
Perceive good location of their home ^b^			**<0.0001**			**<0.0001**			**0.004199**			**<0.0001**
Yes	23%	(22, 25)		11.8	(11.1, 12.5)		13.3	(11.9, 14.9)		4.0	(3.5, 4.6)	
No	48%	(44, 52)		27.8	(25.0, 30.8)		14.9	(12.4, 17.9)		7.2	(5.5, 9.4)	
**Harmful Behaviours**												
Recreational drug use in past 6 months			**<0.0001**			**<0.0001**			0.5917			**0.000258**
Yes	39%	(36, 42)		21.3	(19.4, 23.4)		11.1	(9.1, 13.5)		6.4	(4.8, 8.4)	
No	25%	(24, 27)		11.9	(11.2, 12.6)		12.3	(11.0, 13.7)		4.1	(3.6, 4.8)	
Prior diagnosis of alcohol abuse ^c^			**<0.0001**			**<0.0001**			0.5977			0.2327
Yes	44%	(39, 49)		22.3	(19.7, 25.3)		11.7	(9.3, 14.7)		5.7	(3.5, 9.2)	
No	26%	(24, 27)		12.5	(11.9, 13.2)		12.0	(10.8, 13.3)		4.4	(3.8, 5.0)	
Current smoker			**<0.0001**			**<0.0001**			0.6678			**<0.0001**
Yes	37%	(34, 39)		18.7	(17.5, 20.1)		12.1	(10.6, 13.8)		5.7	(4.7, 7.0)	
No	22%	(20, 24)		10.4	(9.7, 11.2)		11.8	(10.2, 13.5)		3.9	(3.3, 4.6)	
**Health Status**												
History of depression ^d^			**<0.0001**			**<0.0001**			0.5824			
Yes	40%	(38, 43)		20.1	(18.8, 21.5)		12.5	(11.0, 14.1)		—	—	
No	20%	(18, 22)		9.8	(9.1, 10.6)		11.2	(9.6, 13.1)		—	—	
Charlson co-morbidity index ≥ 1			**<0.0001**			**<0.0001**			0.4327			0.7021
Yes	35%	(32, 38)		16.5	(15.0, 18.1)		12.1	(10.1, 14.4)		4.3	(3.3, 5.7)	
No	26%	(24, 27)		12.6	(11.8, 13.3)		11.9	(10.6, 13.4)		4.5	(3.9, 5.2)	
Non-suppressed recent viral loads (> 50 *μL*)(in past 6 months)			**<0.0001**			**<0.0001**			**0.03633**			**<0.0001**
Yes	35%	(32, 38)		19.3	(17.6, 21.1)		7.4	(5.8, 9.4)		5.5	(4.2, 7.2)	
No	25%	(24, 27)		11.9	(11.2, 12.7)		13.6	(12.2, 15.1)		4.2	(3.7, 4.9)	
**Instrument Type** ^e^			**<0.0001**			**<0.0001**			**<0.0001**			**0.001045**
CES-D_20_	22%	(20, 24)		10.0	(9.1, 11.0)		9.8	(8.0, 11.9)		3.6	(2.9, 4.5)	
K_10_	32%	(30, 34)		15.9	(15.0, 16.9)		12.9	(11.5, 14.4)		5.1	(4.4, 6.0)	

CI = confidence intervals, PP = point prevalence, expressed in percentage, PPR = period prevalence rate per 100 person-years, RR = recurrent rate per 100 person-years, IR = incidence rate per 100 person-years

^a^ Difficulty in affording house-related expenses was defined as a participant’s self-reported “Very difficult” or “Fairly difficult” for the following question: *“Considering your household income*, *how difficult is it for you to meet your monthly housing-related costs*?*(Housing costs include rent/mortgage*, *property taxes and utilities only)*.*”*

^b^ A 5-point Likert scale (strongly agree to strongly disagree) was used. We dichotomized their response into “yes” (strongly agree/agree) and “no” (neutral/disagree/strongly disagree).

^c^ Addiction to alcohol was defined as whether HIV-positive participants had a diagnostic code of alcohol dependence/abuse in OHIP (ICD-9: 303) or in main diagnosis of DAD and NACRS (ICD-9-CM: 303; ICD-10-CA: F10), from the earliest available records in these databases to a day before the baseline.

^d^ History of depression was defined as having a past depression-related diagnosis identified in OHIP records (OHIP ICD-9: 296 and 311), from the earliest available records to a year before the baseline.

^e^ There are two instruments for identifying current depressive symptoms administered by clinic nurses and assistant during the participant’s regular clinical appointments. Due to constraints on human resources and time in several HIV clinics, 61% of HIV-positive participants were administered with the 10-item Kessler Psychological Distress Scale (K_10_) and 39% were administrated with the 20-item Centre for Epidemiologic Studies Depression Scale (CES-D_20_). Full details of the cohort can be found on the study website: http://www.ohtncohortstudy.ca/

Our point prevalence was 28% (95% CI: 27–29%) at baseline. By the end of the follow-up period, our period prevalence rate was 4.9 per 100 person-years (95% CI: 4.8–5.1). Consistent results were observed between explanatory variables and both prevalence outcomes (Table 3). For example, more young participants were found to be depressed than participants who were 50 years or older. More women than men were found to be depressed. A higher point prevalence and period prevalence rate of depressive symptoms was found among participants who were: Indigenous, unemployed, disabled, or who had lower educational attainment or low incomes. Higher rates were also found among those who had a history of depression, a history of harmful behaviour, a severe HIV condition or other co-morbidity, and/or poorer housing or neighbourhood conditions.

### Recurrence

We included 957 HIV-positive participants with current depressive symptoms identified at baseline and had at least two-year follow-up data in our recurrence estimation (Fig 2 & Table 3). When we compared the characteristics of participants included in the recurrence sub-cohort to the remainder of the cohort, we found that participants were slightly younger (mean age: 52 v. 55 years; p-value: <0.0001) and more likely to be female (20.9 v. 13.5%; p-value: <0.0001).

By the end of the follow-up period, 43% of participants with current depressive symptoms at baseline had a recurrent episode. The first recurrence rate of current depressive symptoms was 11.9 per 100 person-years (95% CI: 10.8–13.2). The rate peaked with participants who were 50 or older (15.3 per 100 person-years; 95% CI: 13.1–17.9). A higher recurrence rate was found among participants who were European decent or Indigenous, or who were employed or disabled, students or retired, and among those who lived in poorer housing, had supressed recent viral loads, and/or had completed the K_10._

### Incidence

We included 1,745 HIV-positive participants who were depressive symptoms-free at or prior to baseline and who had at least one year of follow-up data for our incidence estimation (Fig 2 & Table 3). When we compared the characteristics of participants included in the incidence sub-cohort to the remainder of the cohort, we found that participants were slightly younger (mean age: 54 v. 55 years; p-value: 0.0019) and more likely to be female (16.1 v. 14.2%; p-value: 0.059).

Among the 1,745 depressive symptoms-free participants, the cumulative incidence of current depressive symptoms was 14% by the end of the follow-up period (Table 3). The incidence rate of current depressive symptoms was 4.5 per 100 person-years (95% CI: 3.9–5.1) and peaked at 16–29 years (7.2 per 100 person-years; 95% CI: 4.7–10.9). Women had a higher incidence rate (6.1 vs. 4.2 per 100 person-years) than men. A higher incidence rate was found among participants who were disabled, recreational drug users, current smokers, separated/divorced/single or widowed, had completed high school or less, had annual household income less than $20K, had non-suppressed recent viral loads, lived in poorer housing, and/or who completed the K_10_.

### Mental Disorder Co-Morbidities

HIV-positive participants with current depressive symptoms had more additional mental disorders compared to their counterparts without these symptoms (3 v. 2, p-value < 0.001). Profiles of other mental disorders by participants’ baseline depressive symptoms status are available in Table 4. In particular, depressed participants were likely to be diagnosed with senile dementia, alcohol psychosis, drug psychosis, schizophrenia, other psychosis, anxiety, personality disorders, alcoholism, drug dependence, tobacco abuse, psychosomatic disturbance, habit spasms, or adjustment reaction. We noted that a high prevalence (78–92%) of anxiety-related diagnosis (ICD-9: 300) among both depressed and non-depressed HIV-positive participants. This might be due to the fact that this diagnostic code is often used by Ontario physicians for a range of anxiety-related symptoms among Ontario population; therefore, the code might overestimate the prevalence of anxiety-related conditions.

**Table 4:** History of Mental Disorders Diagnosis by Current Depressive Symptoms Status at Baseline (N = 3,816).

ICD-9 Mental Disorders Sub-chapters ^a^	ICD-9 ^b^	Current Depressive symptoms		p-value
		With	Without	
		(N = 1070)	(N = 2746)	
Median number of mental disorders diagnosis, (Interquartile range [IQR])		3 (2–4)	2 (1–3)	< 0.0001
Senile dementia, presenile dementia	290	6.0%	3.8%	0.002483
Alcoholic psychosis, delirium tremens, Korsakov’s psychosis	291	2.6%	0.9%	< 0.0001
Drug psychosis	292	0.9%	2.2%	0.0006531
Schizophrenia	295	8.9%	4.1%	< 0.0001
Paranoid states	297	1.5%	1.1%	0.2589
Other psychoses	298	9.1%	4.2%	< 0.0001
Anxiety neurosis, hysteria, neurasthenia, obsessive compulsive neurosis, reactive depression	300	91.6%	78.4%	< 0.0001
Personality disorders	301	18.0%	7.8%	< 0.0001
Sexual deviations	302	3.5%	3.0%	0.4514
Alcoholism	303	19.6%	10.1%	< 0.0001
Drug dependence, drug addiction	304	34.7%	19.5%	< 0.0001
Tobacco abuse	305	13.1%	10.2%	0.01156
Psychosomatic disturbances	306	16.3%	13.5%	0.03145
Habit spasms, tics, stuttering, tension headaches, anorexia nervosa, sleep disorders, enuresis	307	37.0%	26.3%	<0.0001
Adjustment reaction	309	25.5%	17.6%	< 0.0001
Behaviour disorders of childhood and Adolescence	313	3.4%	2.2%	0.04388
Hyperkinetic syndrome of childhood	314	2.1%	1.4%	0.09025

^a^ Mental disorders sub-chapters were defined according to ICD-9 diagnostics codes in the resource manual for physicians

^b^ A modified and abbreviate 3-digit version of the ICD-9 diagnostic codes were adopted for physical billings in the OHIP database.

### Factors Associated with Point Prevalence, Recurrence and Incidence of Current Depressive Symptoms

The results of our multivariable analyses for associations between factors and point prevalence, first recurrence and incidence of current depressive symptoms are presented in Tables 5 to 7.

For point prevalence, we found that depressed participants were more likely to be younger, female and have a history of depression. They were also more likely to have difficulty in affording housing-related expenses, have a history of depression, feel worried about their housing situation, receive government disability subsidies, be unemployed, use recreational drugs, and have completed the K_10_ instrument (Table 5). Participants protected from prevalent depressive symptoms were more likely to have better control of their housing situation, more likely to like their neighbourhood and more likely to have better physical health (Table 5).

**Table 5:** Adjusted Relative Risk (aRR) with 95% Confidence Intervals (CI) for the Explanatory Variables of Point Prevalence of Current Depressive Symptoms among HIV-positive Participants (N = 3,816).

Explanatory Variables	Full Model		Final Model	
	aRR	95% CI	aRR	95% CI
**Demographics**				
Age				
16–29 years	**1.35**	**(1.04, 1.75)**	**1.36**	**(1.06, 1.76)**
30–39 years	**1.35**	**(1.13, 1.61)**	**1.36**	**(1.14, 1.63)**
40–49 years	**1.23**	**(1.07, 1.42)**	**1.25**	**(1.09, 1.44)**
≥ 50 years (reference)	1			
Gender				
Female	**1.22**	**(1.07, 1.66)**	**1.20**	**(1.02, 1.40)**
Male (reference)	1		1	
Sexual orientation				
Gay, lesbian, or bisexual	1.01	(0.87, 1.16)	0.99	(0.86, 1.14)
Heterosexual (reference)	1		1	
Marital status				
Married / living with partners	0.89	(0.78, 1.02)	0.88	(0.77, 1.00)
Single, separated/divorced, or widowed (reference)	1		1	
Ethnic identity				
First Nation, Metis, or Inuit	1.02	(0.86, 1.21)	1.04	(0.87, 1.23)
African, Caribbean, Asian, or Latin American	1.26	(0.99, 1.60)	1.25	(0.99, 1.59)
European descent (reference)	1		1	
Immigration status				
Canadian immigrant	1.13	(0.99, 1.30)	1.11	(0.97, 1.27)
Canadian born (reference)	1		1	
**Socio-economic Status**				
Current employment status				
Unemployed	**1.33**	**(1.07, 1.66)**	**1.32**	**(1.07, 1.64)**
Student/Retired	0.85	(0.62, 1.17)	0.83	(0.61, 1.14)
Recipient of Ontario Disability Support Program	**1.43**	**(1.20, 1.70)**	**1.42**	**(1.20, 1.69)**
Employed (reference)	1		1	
Education attainment				
Completed high school or less	1.01	(0.90, 1.14)	—	—
More than high school (reference)	1			
Annual household income (CAD) before withholding taxes/benefits				
< $20,000	1.00	(0.82, 1.22)	1.03	(0.85, 1.25)
$20,000 to $39,999	1.06	(0.87, 1.28)	1.08	(0.89, 1.30)
$40,000 to $49,999	0.93	(0.74, 1.17)	0.97	(0.77, 1.21)
≥ $50,000 (reference)	1		1	
**Housing and Neighbourhood Conditions**				
Difficulty in affording housing-related expenses ^a^				
Yes	**1.21**	**(1.07, 1.38)**	**1.21**	**(1.07, 1.37)**
No (reference)	1		1	
Worry about being force out from their home ^b^				
Yes	**1.32**	**(1.17, 1.50)**	**1.31**	**(1.16, 1.48)**
No (reference)	1		1	
Having control over their housing situation ^b^				
Yes	**0.83**	**(0.73, 0.95)**	**0.84**	**(0.73, 0.95)**
No (reference)	1		1	
**Sense of** belonging to neighbourhood ^b^				
Yes	0.89	(0.79, 1.02)	0.89	(0.79, 1.02)
No (reference)	1		1	
Perceive good location of their home ^b^				
Yes	**0.84**	**(0.73, 0.95)**	**0.83**	**(0.73, 0.96)**
No (reference)	1		1	
**Harmful Behaviours**				
Recreational drug use in past 6 months				
Yes	**1.21**	**(1.06, 1.37)**	**1.21**	**(1.07, 1.38)**
No (reference)	1		1	
Prior diagnosis of alcohol abuse ^c^				
Yes	1.03	(0.89, 1.20)	—	—
No (reference)	1			
Current smoker				
Yes	1.10	(0.97, 1.25)	—	—
No (reference)	1			
**Health Status**				
History of depression ^d^				
Yes	**1.48**	**(1.31, 1.67)**	**1.50**	**(1.33, 1.69)**
No (reference)	1		1	
Physical component of SF-12 (Increased by every five points)				
Physical functioning	1.00	(0.96, 1.03)	—	—
Bodily pain	**0.97**	**(0.94, 0.99)**	**0.97**	**(0.94, 0.99)**
Role functioning	**0.89**	**(0.86, 0.93)**	**0.89**	**(0.87, 0.93)**
General health	**0.91**	**(0.89, 0.94)**	**0.91**	**(0.89, 0.93)**
Charlson co-morbidity index ≥ 1				
Yes	0.91	(0.80, 1.02)	—	—
No (reference)	1			
Non-suppressed recent viral loads (> 50 *μL*) (in past 6 months)				
Yes	1.07	(0.940, 1.21)	1.07	(0.95, 1.21)
No (reference)	1		1	
Years since HIV diagnosis (Increased by a year)	1.00	(0.99, 1.01)	1.00	(0.99, 1.01)
**Instrument type** ^e^				
K_10_	**1.30**	**(1.17, 1.48)**	**1.32**	**(1.17, 1.49)**
CES-D_20_ (reference)	1		1	

Footnotes: This table contains the full model (with all explanatory variables) and the final set of explanatory variables retained in the multivariable modified Poisson regression models with sandwich variance estimators for point prevalence of current depressive symptoms.

^a^ Difficulty in affording house-related expenses was defined as a participant’s self-reported “Very difficult” or “Fairly difficult” for the following question: *“Considering your household income*, *how difficult is it for you to meet your monthly housing-related costs*?*(Housing costs include rent/mortgage*, *property taxes and utilities only)*.*”*

^b^ A 5-point Likert scale (strongly agree to strongly disagree) was used. We dichotomized their response into “yes” (strongly agree/agree) and “no” (neutral/disagree/strongly disagree).

^c^ Addiction to alcohol was defined as whether HIV-positive participants had a diagnostic code of alcohol dependence/abuse in OHIP (ICD-9: 303) or in main diagnosis of DAD and NACRS (ICD-9-CM: 303; ICD-10-CA: F10), from the earliest available records in these databases to a day before the baseline.

^d^ History of depression was defined as having a past depression-related diagnosis identified in OHIP records (OHIP ICD-9: 296 and 311), from the earliest available records to a year before the baseline.

^e^ There are two instruments for identifying current depressive symptoms administered by clinic nurses and assistant during the participant’s regular clinical appointments. Due to constraints on human resources and time in several HIV clinics, 61% of HIV-positive participants were administered with the 10-item Kessler Psychological Distress Scale (K_10_) and 39% were administrated with the 20-item Centre for Epidemiologic Studies Depression Scale (CES-D_20_). Full details of the cohort can be found on the study website: http://www.ohtncohortstudy.ca/

For first recurrence, our multivariable model was stratified by instrument type and recreational drug use because these variables did not meet the proportionality assumptions. Participants with greater risk of first recurrent depressive symptoms were more likely to feel worried about their housing situation (Table 6). We noted that participants with a history of depression were more likely to have recurrent depressive symptoms at a borderline statistically significant level (p-value = 0.082) (Table 6). Participants with better physical health or non-suppressed viral loads were less likely to have recurrent depressive symptoms (Table 6).

**Table 6:** Adjusted Hazard Ratio (aHR) with 95% Confidence Intervals (CI) for Explanatory Variables of the First Recurrence of Current Depressive Symptoms among HIV-positive Participants (N = 957).

Explanatory Variables	Full Model		Final Model	
	aHR	95% CI	aHR	95% CI
**Demographics**				
Age				
16–29 years	0.96	(0.47, 1.96)	1.05	(0.56, 1.95)
30–39 years	0.99	(0.66, 1.49)	0.98	(0.68, 1.41)
40–49 years	1.18	(0.90, 1.54)	1.26	(0.98, 1.61)
≥ 50 years (reference)	1			
Gender				
Female	0.98	(0.67, 1.43)	0.98	(0.73, 1.30)
Male (reference)	1		1	
Sexual orientation				
Gay, lesbian, or bisexual	0.98	(0.71, 1.38)	—	—
Heterosexual (reference)	1			
Marital status				
Married / living with partners	1.00	(0.77, 1.32)	—	—
Single, separated/divorced, or widowed (reference)	1			
Ethnic identity				
First Nation, Metis, or Inuit	0.86	(0.59, 1.26)	—	—
African, Caribbean, Asian, or Latin American	0.55	(0.25, 1.23)	—	—
European descent (reference)	1			
Immigration status				
Canadian immigrant	1.25	(0.94, 1.66)	1.29	(1.00, 1.66)
Canadian born (reference)	1		1	
**Socio-economic Status**				
Current employment status				
Unemployed	0.96	(0.57, 1.62)	**—**	**—**
Student/Retired	1.17	(0.68, 2.01)	—	—
Recipient of Ontario Disability Support Program	0.91	(0.68, 1.21)	**—**	**—**
Employed (reference)	1			
Education attainment				
Completed high school or less	1.07	(0.82, 1.39)	—	—
More than high school (reference)	1			
Annual household income (CAD) before withholding taxes/benefits				
< $20,000	1.02	(0.69, 1.50)	0.99	(0.72, 1.36)
$20,000 to $39,999	0.96	(0.65, 1.41)	0.94	(0.67, 1.33)
$40,000 to $49,999	0.73	(0.45, 1.17)	0.72	(0.46, 1.12)
≥ $50,000 (reference)	1		1	
**Housing and Neighbourhood Conditions**				
Difficulty in affording housing-related expenses ^a^				
Yes	1.13	(0.87, 1.45)	**—**	**—**
No (reference)	1			
Worry about being force out from their home ^b^				
Yes	**1.37**	**(1.05, 1.78)**	**1.49**	**(1.17, 1.90)**
No (reference)	1		1	
Having control over their housing situation ^b^				
Yes	0.85	(0.65, 1.12)	**—**	**—**
No (reference)	1			
**Sense of** belonging to neighbourhoods ^b^				
Yes	—	—	—	—
No (reference)				
Perceive good location of their home ^b^				
Yes	1.00	(0.74, 1.37)	**—**	**—**
No (reference)	1			
**Harmful Behaviours**				
Recreational drug use in past 6 months				
Yes	**—**	**—**	**—**	**—**
No (reference)				
Prior diagnosis of alcohol abuse ^c^				
Yes	0.91	(0.65, 1.28)	—	—
No (reference)	1			
Current smokers				
Yes	1.05	(0.82, 1.35)	—	—
No (reference)	1			
**Health Status**				
History of depression ^d^				
Yes	1.23	(0.96, 1.57)	1.23	(0.97, 1.54)
No (reference)	1		1	
Use of antidepressant (in past 12 months) ^e^				
Yes	1.13	(0.84, 1.51)	1.09	(0.82, 1.44)
No (reference)	1		1	
Physical component of SF-12 (Increased by every five points)				
Physical functioning	0.95	(0.89, 1.02)	0.95	(0.89, 1.02)
Bodily pain	1.04	(0.99, 1.10)	1.04	(0.98, 1.09)
Role functioning	**0.88**	**(0.81, 0.95)**	**0.88**	**(0.82, 0.95)**
General health	**0.94**	**(0.89, 0.99)**	**0.94**	**(0.89, 0.99)**
Charlson co-morbidity index ≥ 1				
Yes	1.01	(0.77, 1.30)	—	—
No (reference)	1			
Non-suppressed recent viral loads (> 50 *μL*) (in past 6 months)				
Yes	1.01	(0.78, 1.31)	—	—
No (reference)	1			
Years since HIV diagnosis (Increased by every year)	0.99	(0.98, 1.01)	—	—
**Instrument type** ^f^				
K_10_	**2.24**	**(1.66, 3.03)**	**—**	**—**
CES-D_20_ (reference)	1			

This table contains the full model (with all explanatory variables) and the final set of explanatory variables retained in the multivariable Cox proportional hazard regression models for the first recurrence of current depressive symptoms. The full Cox proportional hazard model was stratified by sense of belonging to their neighbourhoods and recreational drug use because these variables did not satisfy proportionality assumption. The final Cox proportional hazard model was stratified by instrument type and recreational drug use because these variables did not satisfy proportionality assumption.

^a^ Difficulty in affording house-related expenses was defined as a participant’s self-reported “Very difficult” or “Fairly difficult” for the following question: *“Considering your household income*, *how difficult is it for you to meet your monthly housing-related costs*?*(Housing costs include rent/mortgage*, *property taxes and utilities only)*.*”*

^b^ A 5-point Likert scale (strongly agree to strongly disagree) was used. We dichotomized their response into “yes” (strongly agree/agree) and “no” (neutral/disagree/strongly disagree).

^c^ Addiction to alcohol was defined as whether HIV-positive participants had a diagnostic code of alcohol dependence/abuse in OHIP (ICD-9: 303) or in main diagnosis of DAD and NACRS (ICD-9-CM: 303; ICD-10-CA: F10), from the earliest available records in these databases to a day before the baseline.

^d^ History of depression was defined as having a past depression-related diagnosis identified in OHIP records (OHIP ICD-9: 296 and 311), from the earliest available records to a year before the baseline.

^e^ The definition of antidepressants was based on the first line of antidepressants for managing depression in adults recommended by the Canadian Network for Mood and Anxiety Treatments (CANMAT) Clinical guidelines (Lam et al., 2009)

^f^ There are two instruments for identifying current depressive symptoms administered by clinic nurses and assistant during the participant’s regular clinical appointments. Due to constraints on human resources and time in several HIV clinics, 61% of HIV-positive participants were administered with the 10-item Kessler Psychological Distress Scale (K_10_) and 39% were administrated with the 20-item Centre for Epidemiologic Studies Depression Scale (CES-D_20_). Full details of the cohort can be found on the study website: http://www.ohtncohortstudy.ca/

For incidence, our multivariable model was stratified by general health quality-of-life because this variable did not meet the proportionality assumptions. We found that participants at risk of developing current depressive symptoms were likely to be younger, gay or bisexual, unable to afford housing-related expenses, feel worried about eviction, or use recreational drug (Table 7). Participants with better physical health who liked their neighbourhoods and were, married or living with partners were less likely to have incident depressive symptoms (Table 7).

**Table 7:** Adjusted Hazard Ratios (aHR) with 95% Confidence Intervals (CI) for the Explanatory Variables of Incidence of Current Depressive Symptoms among HIV-positive Patients Participants (N = 1,745).

Explanatory Variables	Full Model		Final Model	
	aHR	95% CI	aHR	95% CI
**Demographics**				
Age				
16–29 years	1.94	(0.97, 3.86)	**2.34**	**(1.35, 4.04)**
30–39 years	**1.70**	**(1.06, 2.73)**	**1.96**	**(1.31, 2.93)**
40–49 years	1.43	(0.99, 2.06)	**1.56**	**(1.13, 2.16)**
≥ 50 years (reference)	1		1	
Gender				
Female	1.07	(0.62, 1.84)	1.32	(0.84, 2.09)
Male (reference)	1		1	
Sexual orientation				
Gay, lesbian, or bisexual	1.55	(0.98, 2.46)	**1.49**	**(1.02, 2.19)**
Heterosexual (reference)	1		1	
Marital status				
Married / living with partners	0.72	(0.52, 1.01)	**0.69**	**(0.52, 0.91)**
Single, separated/divorced, or widowed (reference)	1		1	
Ethnic identity				
First Nation, Metis, or Inuit	1.26	(0.76, 2.11)	1.24	(0.81, 1.92)
African, Caribbean, Asian, or Latin American	2.02	(0.75, 5.41)	2.06	(0.88, 4.82)
European descent (reference)	1		1	
Immigration status				
Canadian immigrant	1.03	(0.71, 1.50)	—	—
Canadian born (reference)	1			
**Socio-economic Status**				
Current employment status				
Unemployed	1.33	(0.69, 2.59)	—	—
Student/Retired	0.77	(0.41, 1.46)	—	—
Recipient of Ontario Disability Support Program	1.11	(0.76, 1.63)	—	—
Employed (reference)	1			
Educational attainment				
Completed high school or less	1.13	(0.80, 1.60)	—	—
More than high school (reference)	1			
Annual household income (CAD) before withholding taxes/benefits				
< $20,000	1.39	(0.85, 2.26)	—	—
$20,000 to $39,999	1.1	(0.69, 1.75)	—	—
$40,000 to $49,999	1.37	(0.85, 2.20)	—	—
≥ $50,000 (reference)	1			
**Housing and Neighbourhood Conditions**				
Difficulty in affording housing-related expenses ^a^				
Yes	**1.53**	**(1.07, 2.19)**	**1.60**	**(1.17, 2.18)**
No (reference)	1			
Worry about being force out from their home ^b^				
Yes	1.34	(0.90, 1.98)	**1.64**	**(1.17, 2.28)**
No (reference)	1			
Having control over their housing situation ^b^				
Yes	0.72	(0.49, 1.07)	—	—
No (reference)	1			
**Sense of** belonging to the neighbourhoods ^b^				
Yes	0.82	(0.57, 1.18)	—	—
No (reference)	1			
Perceive good location of their home ^b^				
Yes	**—**	**—**	**0.56**	**(0.40, 0.78)**
No (reference)	1			
**Harmful Behaviours**				
Recreational drug use in past 6 months				
Yes	**1.74**	**(1.17, 2.58)**	**1.52**	**(1.07, 2.17)**
No (reference)	1		1	
Prior diagnosis of alcohol abuse ^c^				
Yes	0.8	(0.44, 1.47)	—	—
No (reference)	1			
Current smoker				
Yes	1.08	(0.78, 1.51)	—	—
No (reference)	1			
**Health Status**				
History of depression ^d^				
Yes	**1.48**	**(1.31, 1.67)**	—	—
No (reference)	1			
Physical component of SF-12 (Increased by every five points)				
Physical functioning	1	(0.90, 1.09)	—	—
Bodily pain	0.93	(0.87, 1.01)	**0.93**	**(0.87, 0.99)**
Role functioning	**0.88**	**(0.80, 0.98)**	**0.88**	**(0.82, 0.95)**
General health	**0.78**	**(0.73, 0.84)**	—	—
Charlson co-morbidity index ≥ 1				
Yes	**0.56**	**(0.38, 0.84)**	**0.71**	**(0.50, 0.99)**
No (reference)	1			
Non-suppressed recent viral loads (> 50 *μL*) (in past 6 months)				
Yes	0.85	(0.57, 1.29)	1.04	(0.73, 1.47)
No (reference)	1		1	
Years since HIV diagnosis (Increased by every year)	0.99	(0.97, 1.02)	—	—
**Instrument type** ^e^				
K_10_	1.31	(0.94, 1.83)	**1.37**	**(1.02, 1.84)**
CES-D_20_ (reference)	1		1	

Footnotes: This table contains the full model (with all explanatory variables) and the final set of explanatory variables retained in the multivariable Cox proportional hazard models for incidence of current depressive symptoms. The full Cox proportional hazard model was stratified by perception of good location of participants’ home because this variable did not satisfy proportionality assumption. The final Cox proportional hazard model was stratified by general health quality-of-life because this variable did not satisfy proportionality assumption.

^a^ Difficulty in affording house-related expenses was defined as a participant’s self-reported “Very difficult” or “Fairly difficult” for the following question: *“Considering your household income*, *how difficult is it for you to meet your monthly housing-related costs*?*(Housing costs include rent/mortgage*, *property taxes and utilities only)*.*”*

^b^ A 5-point Likert scale (strongly agree to strongly disagree) was used. We dichotomized their response into “yes” (strongly agree/agree) and “no” (neutral/disagree/strongly disagree).

^c^ Addiction to alcohol was defined as whether HIV-positive participants had a diagnostic code of alcohol dependence/abuse in OHIP (ICD-9: 303) or in main diagnosis of DAD and NACRS (ICD-9-CM: 303; ICD-10-CA: F10), from the earliest available records in these databases to a day before the baseline.

^d^ History of depression was defined as having a past depression-related diagnosis identified in OHIP records (OHIP ICD-9: 296 and 311), from the earliest available records to a year before the baseline.

^e^ There are two instruments for identifying current depressive symptoms administered by clinic nurses and assistant during the participant’s regular clinical appointments. Due to constraints on human resources and time in several HIV clinics, 61% of HIV-positive participants were administered with the 10-item Kessler Psychological Distress Scale (K_10_) and 39% were administrated with the 20-item Centre for Epidemiologic Studies Depression Scale (CES-D_20_). Full details of the cohort can be found on the study website: http://www.ohtncohortstudy.ca/

## Discussion

To our knowledge, this is the first study describing the prevalence, recurrence and incidence of current depressive symptoms systematically and longitudinally among people living with HIV receiving care in Ontario, Canada. We found that the prevalence, recurrence, and incidence of current depressive symptoms in HIV-positive participants are high. Approximately 28% of HIV-positive participants were identified with current depressive symptoms, and the cumulative incidence was approximately 14%. Our results also revealed that depressive symptoms in HIV-positive participants was likely to be chronic and recurrent. Of those with a prevalent case at baseline, 43% had a recurrent depressive episode during our five-year follow-up period. Elevated point prevalent and rates of recurrent or incident depressive symptoms were statistically significant among individuals with a history of depression and among those who were: female; younger; lesbian, gay, or bisexual; living in poorer housing conditions; recreational drug users; unemployed; or receiving government disability subsidies.

In Ontario, Canada, our study is the first to provide comprehensive evidence of the burden of depression and the underlying catalysts of the condition from a longitudinal perspective. This is an important first step to inform health care providers, policy-makers, and program planners when making decisions and planning for evidence-based mental health care and support for people living with HIV in Ontario. In North America, although in past decades, efforts were made to coordinate and integrate mental health care and support in HIV care, unmet needs in mental health care and services still remain a major challenge for these individuals—half of people living with HIV are under-recognized for their depressive symptoms condition, and only half are being treated [6–9,67,68]. Several studies have revealed that mental health care in the current HIV model is not well-coordinated and integrated [69–71]. A recent U.S. study showed that depression is not regularly monitored in people living with HIV despite the high prevalence of depression in this population—only 31% of 72 HIV care providers from large academic medical centres routinely assess depression for people living with HIV, and only 13% follow-up with their patients within 2 weeks after prescribing an antidepressant [69]. There are many challenges to delivering regular care for depression within current HIV care model. Curran et al. (2011) conducted a qualitative study with eight people living with HIV, seven mental health providers, and 18 HIV health care providers at the U.S. Veterans Affairs HIV clinics. The researchers revealed that there are time constraints in dealing with depression along with other complex health conditions during appointments [70]. Some providers are worried about drug interactions between combination antiretroviral therapy (cART) and antidepressants, and others had difficulty referring their patients to mental health specialists [70]. Some providers found it difficult to diagnose depression because of the similarity between depressive symptoms and HIV symptoms; and others did not feel that they had the expertise necessary to treat depression in these patients [70]. Our findings of the high point prevalence and recurrence rate of depressive symptoms in people living with HIV reinforce the importance of effective delivery of mental health care in the context of HIV treatment and demonstrate the need for long-term support and routine management of depression, particularly among those at high risk.

When compared to prior studies, our results on the point prevalence of current depressive symptoms (28%) are comparable to recent findings (23–26%) from large-scale studies conducted in the U.S. [58,72,73]. However, our results showed a lower recurrence rate of 43% when compared to the 61% rate found by Malee et al.(2014) [39] and to the 90% rate found by an older study conducted by Johnson and colleagues (1999) [40]. This difference might due to the fact that Malee’s sample was restricted to HIV-positive females and Johnson’s sample was restricted to injection drug users; these participants are more likely to develop depressive symptoms. Additionally, our definition of “recurrent depressive symptoms” used a two-year window from baseline while Malee et al used one-year window and Johnson et al. used a six-month window. Furthermore, our study focused on current depressive symptoms alone while the Malee study examined a number of psychiatric disorders.

Our cumulative incidence of depressive symptoms of 14% during follow-up among a depressive symptoms-free cohort at baseline was lower than cumulative incidence of 21% reported in the Malee study [39]. Our incidence rate of 4.5 per 100 person-years was similar to the rate of 3.9 per 100 person-years reported by Anagnostopoulos et al. in a depressive symptoms -free sample of the Swiss HIV Cohort [38]. However, our incidence rate was higher than prior findings of 1.04–2.2 per 100 person-years reported in French and French Guiana studies [35–37] where their denominator included all participants and that would underestimate the incidence rate.

Consistent with prior studies [38,58–61], we found an elevated point prevalence of depressive symptoms among HIV-positive participants who had a history of depression and among those who were: female, younger, recreational drug users, or unemployed/disabled. Unlike the U.S. evidence [58,61], we did not note differences among ethnocultural minorities. This might due to 84% of our sample being Caucasian. Unlike prior studies [58,74], we did not find that being lesbian, gay, or bisexual was associated with an elevated point prevalence of depressive symptoms; however, we did find those who self-reported as lesbian, gay, or bisexual were about two times more likely to develop incident depressive symptoms during follow-up.

Our study is the first to provide further examination on factors associated with recurrent depressive symptoms in people living with HIV. Although Malee et al. also explored factors associated to recurrent depressive symptoms, this study was limited to specific factors related to HIV-positive mothers (e.g. single parenting, functional limitations for being a caregiver, smoking during pregnancy, etc.) [39]. Additionally, Malee et al. also did not consider history of depression in their model [39]. Consistent with a systematic review focusing on the general population [75], our results indicate that a history of depression was associated with recurrent depressive symptoms, although it was found at a borderline statistically significant level (p-value = 0.082). The borderline level might due to the use of diagnostic codes in physicians’ billing records for identifying a history of depression, which might contribute to some degree of misclassification [76].

With regard to factors associated with incidence rate of depressive symptoms, consistent with prior evidence, we found HIV-positive participants who were younger or recreational drug users were more likely to develop incident depressive symptoms [35–38]. Unlike prior evidence [35,37], we did not find that those with more severe HIV conditions were more likely to develop incident depressive symptoms but we did observe the incident cases were associated with poor physical health. Additionally, we noted that the HIV-positive participants with other physical comorbidities were less likely to develop incident depressive symptoms; however, these participants might be sicker and might be more likely to die before the incident cases occur [13].

Our study is the first to report associations between prevalent, recurrent and incident depressive symptoms and an extensive number of housing and neighbourhood-related contextual factors in people living with HIV. Cross-sectional evidence has shown that poorer and unstable housing conditions are strong explanatory variables of poor mental health outcomes among these people living with HIV [77,78]. Our study further explored causal relationship between housing and neighbourhood-related factors and incidence and recurrence of depressive symptoms. HIV-positive participants who felt worried about their housing situation were more likely to have elevated recurrence rate. HIV-positive participants who had difficulty in affording housing-related expenses, or who felt worried about their housing situation were more likely to have higher rate of incident depressive symptoms whereas those who perceived good location of their home were protected from developing incident depression. Our results from a longitudinal perspective strengthen the current knowledge that poorer housing and neighbourhood conditions may worsen depressive symptoms in people living with HIV in the long run. In North America, people living with HIV are struggling with poverty and unstable housing situations [23,43]. These results also reinforce the importance of providing stable and good quality neighbourhood conditions in current HIV policy and programs, which may help alleviate depressive symptoms and improve the overall well-being of people living with HIV over time.

Our study has several strengths. First, the OCS is the largest HIV cohort in Ontario, representing about one-fifth of the HIV-positive population in the province; participant characteristics generally represent typical HIV-positive individuals in care [43]. Second, this is the first study to provide comprehensive information about depressive symptoms and its underlying associated catalysts among people living with HIV from a longitudinal perspective. Third, our use of linked data between the OCS (prospective cohort study) and administrative databases overcomes limitations associated with using a single dataset. Fourth, the current study is one of the first large-scale HIV cohort studies to demonstrate an association between housing and neighbourhood factors and prevalent, recurrent, and incident depressive symptoms in Canada.

Our study has some limitations. First, we relied on screening instruments (i.e., the CES-D_20_ and K_10_), to identify current depressive symptoms, but excellent agreement between these instruments and DSM-IV-TR criteria (for major depression diagnosis) have been demonstrated in this cohort (Sensitivity: 0.97–1.0; Specificity: 0.81–0.87) [46]. The two instruments also demonstrated good interrater agreement when compared against DSM-IV-TR criteria. Second, although our data sources are comprehensive, some important explanatory variables—including childhood adversity, stigma and coping strategies—were not included in our analysis. Third, our recurrence and incidence rates might be underestimated because there were gaps between our measures of depressive symptoms; similarly, the rates might be overestimated because participants were followed prospectively, possibly leaving the HIV-positive participants with more complex medical needs in the study. Fourth, we relied on depression-related diagnostic codes from participants’ health service utilization records to identify an eight-week depression-free period when estimating recurrent depressive symptoms rate. However, health service utilization records were dependent on whether the HIV-positive participants sought help from physicians, frequency of their doctor appointments, or how well the participants can access health services. Future research should verify our results of recurrent rate using more accurate depressive symptoms measures. Additionally, misclassification is possible—a validation study showed that although depressive symptoms-related diagnostic codes have good positive (>89%) and negative (>91%) predictive values for identifying depressive symptoms, sensitivity of these codes were low (28–35%) [76].

## Conclusions

Despite these limitations, the linked data between the OCS and the administrative databases offer useful new information in understanding the epidemiology of depressive symptoms in HIV-positive participants from a longitudinal perspective—current depressive symptoms is highly prevalent and is likely to recur over time, particularly in some high-risk subgroups. Our results support the direction of Ontario’s HIV/AIDS Strategy to 2026, which addresses medical concerns associated with HIV (such as depression) and the social drivers of health in order to enhance the overall well-being of people living with or at risk of HIV. Our findings reinforce the importance of providing effective mental health care and demonstrate the need for long-term support and routine management of depression, particularly for individuals at high risk.

## Supporting Information

S1 Appendix Measurements of Explanatory Variables. (DOCX)
