# Supplementary material for: Prevalence, Recurrence, and Incidence of Current Depressive Symptoms among People Living with HIV in Ontario, Canada: Results from the Ontario HIV Treatment Network Cohort Study
Source: PLoS One. 2016 Nov 1;11(11):e0165816. doi: 10.1371/journal.pone.0165816 (PMC5089724; doi:10.1371/journal.pone.0165816)
Supplement: S1 Appendix — (DOCX) [file pone.0165816.s001.docx]

**S1 Appendix. Measurements of Explanatory Variables**

1. **Demographics**
2. Participants’ age was derived from their birth date and each interview date. Age was categorised into four groups (i.e. 16-29, 30-39, 40-49, or ≥50 years).
3. Participants’ gender was categorised into male and female.
4. Participants’ self-report sexual orientation was categorised as lesbian, gay, or bisexual; or heterosexual.
5. Participants’ self-report ethnicity was categorised into three groups (Aboriginal; African/Caribbean, Asian, Latin American; or European descent).
6. Participants’ self-report immigration status was categorised as Canadian immigrants or Canadian born.
7. **Socio-economic status**
8. Participants’ annual gross household income (before withholding taxes and benefits) was categorised into four groups (<$20K, $20K-$39,999, $40K-$49,999, or ≥$50K CAD).
9. Participants’ employment status was categorised into four groups (employed; unemployed or not in workforce; student or retired; current recipients of Ontario Disability Support Program).
10. Participants’ educational attainment was categorised into two groups (completed high school or less; more than high school education).
11. Participants’ marital status was categorized into two groups: being married or living with living with partners; or single, separated or divorced, or widowed.
12. **Housing and neighbourhoods condition**

We used a 5-point Likert scale (strongly agree to strongly disagree) to measure participants’ perception on their housing and neighbourhoods condition

1. Participants had difficulty in affording housing-related expense if they responded

“Strongly agree or agree” to the following question: “Considering your household income, how difficult is it for you to meet your monthly housing-related costs?”

1. Participants felt worried about being forced to move out if they responded

“Strongly agree or agree” to the following statement: “I often worry about being forced to move out of my current home”.

1. Participants had control in their housing situation if they responded

“Strongly agree or agree” to the following statement: “At home, I feel I have control over what happens in most situations”.

1. Participants felt belong to their neighbourhoods if they responded

“Strongly agree or agree” to the following statement: “I feel like I belong in my neighborhood”.

1. Participants perceived good location of their home if they responded “Strongly agree or agree” to the following statement: “My home provides a good location for me to live my life.”
2. **Harmful Behaviors**
   1. Current smoking status (yes/no) was self-reported by participants.
   2. Participants’ history of alcohol abuse was determined by whether they had a diagnostic code of alcohol dependence/abuse in Ontario Health Insurance Plan (OHIP) claim database (ICD-9: 303) or in main diagnosis of Discharge Abstract database (DAD) and NACRS (ICD-9-CM: 303; ICD-10-CA: F10), from the earliest available records in these databases to a day before the baseline.
   3. Recreational drug use (in past 6 months) (yes/no) was self-reported by participants
3. **Health Status**
   1. Participants’ history of depression was determined by whether they had an International Statistical Classification of Diseases and Related Health Problems 9th revision (ICD-9) or 10th revision (ICD-10) diagnostic code (Table 1) in the OHIP, DAD or NACRS databases from the earliest available record until one year prior to baseline.
   2. Participants were identified with non-suppressed recent viral loads (in past 6 months) if they had their recent HIV viral load/HIV antigen tests with viral loads (>50$\mu L$) during past 6 months from each interview date.
   3. We derived years since HIV diagnosis for participants using their date of HIV diagnosis and each interview date.
   4. We used twelve-item short form health survey version 2 (SF-12v2) (Chariyalertsak et al., 2011; Ware, Kosinski, & Keller, 1996) to measure participants’ physical health-related quality of life. We also examined four sub-domains of physical quality of life including bodily pain, physical functioning, role physical functioning, and general health.
   5. We used Charlson-Deyo comorbidity index (Deyo, Cherkin, & Ciol, 1992) to measure multi-morbidity of the participants. We used the index of greater than 1 to indicate multi-morbidity.
